# Prevalence of Personality Disorders in Cancer Patients: A Systematic Literature Review in the Era of Personalized Cancer Care

**DOI:** 10.3390/cancers17243901

**Published:** 2025-12-05

**Authors:** Paola Arnaboldi, Ilaria Massari, Elisabetta Lombardi, Marco Cavallo

**Affiliations:** 1 Department of Theoretical and Applied Sciences, eCampus University, Via Isimbardi 10, 22060 Novedrate, Italy; elisabetta.lombardi@uniecampus.it (E.L.); marco.cavallo@uniecampus.it (M.C.); 2 Medical Psychiatry and Medical Psychology Service, Via Tesserete 67, 6942 Savosa, Switzerland; ilaria.massari@hsn.ti.ch; 3 Ticino Cancer League, Piazza Nosetto 3, 6500 Bellinzona, Switzerland

**Keywords:** cancer, personality disorders, prevalence, psycho-oncology

## Abstract

Background: Psychosocial oncology research has largely focused on DSM Axis I disorders such as depression and anxiety, while broader psychological dimensions—particularly personality functioning—have received limited attention. In the context of long-term survivorship and chronic cancer care, understanding personality disorders (PDs) is increasingly important. Objective: This systematic review aimed to estimate the prevalence of PDs in adults with cancer based on studies using DSM or ICD diagnostic frameworks. Methods: Following PRISMA guidelines, we systematically searched major electronic databases (PubMed, Scopus, Web of Science) for studies published over the past 45 years that reported PD prevalence in individuals with cancer. Eligible studies employed DSM or ICD criteria for diagnosing PDs. Screening, data extraction, and quality assessments were conducted independently by multiple reviewers. Extracted data included study design, sample characteristics, diagnostic tools, and PD prevalence. Results: Seven studies met the inclusion criteria. Reported prevalence estimates varied widely, ranging from 1.4% to 100%. Most studies were characterized by small sample sizes, cross-sectional designs, and a primary focus on breast cancer populations; only one study included a broader cancer cohort. Study quality was generally low to moderate. A major limitation across studies was the use of unvalidated or non-standardized diagnostic instruments, along with limited methodological detail and heterogeneity in assessment approaches, preventing meaningful pooling of results. Conclusions: The findings reveal a substantial lack of high-quality research examining personality disorders in oncology settings. Robust, methodologically rigorous studies using validated diagnostic tools are urgently needed to clarify the prevalence and clinical relevance of PDs in cancer populations and to inform the development of comprehensive, patient-centered psychosocial care models.

## Simple Summary

Cancer is increasingly becoming a curable disease thanks to life-saving treatments, which are often long, demanding, and affect many aspects of patients’ lives. These changes challenge the scientific and medical communities to find better ways to care for patients through teamwork among different professionals. From a psychological point of view, there is a need to move from focusing only on symptoms to understanding each patient’s personality functioning. This systematic review aims to fill a gap in existing research by analyzing how common personality disorders are among people with cancer, based on internationally recognized diagnostic criteria. The findings support the development of a new, more holistic model of care that reflects the complexity of both patients and healthcare systems.

## 1. Introduction

Cancer has significantly evolved as a disease, transforming from a primarily fatal condition to one that includes a long-lasting chronic phase [1]. In light of this evidence, the traditional paradigm of care is no longer sufficient to meet the needs of patients or to fully grasp the complexities faced by healthcare personnel in caring for them. These challenges include managing the emotional burden and psychological sequelae of prolonged, demanding treatments, communicating bad news, and addressing chronic pain and fatigue [2,3].

The previous paradigm viewed cancer primarily as a life-threatening disease with a short survival time [4]. From a psychological perspective, most efforts from healthcare personnel, patients, and their families were focused on coping with death anxiety. During this intense phase of the patient’s life, any difficult behavior was often understood and accepted with compassion as a normal reaction to the overwhelming threat of death. This was a symptom-focused paradigm, with psychological interventions aimed mainly at the treatment of symptoms. Consistent with this perspective, psycho-oncology research has predominantly focused on the prevalence of anxiety and depressive symptoms, or on other symptom-based diagnoses [5,6,7,8] as described by the diagnostic and statistical manual (DSM) of mental disorders, Axis I [9,10,11].

The prevalence of personality disorders has often been overlooked. For instance, in one of the most relevant studies that examined the four-week prevalence of mental disorders in cancer patients across major tumor entities using DSM-IV criteria, personality disorders were not considered at all [7].

The study by Minagawa [12], which is particularly important for its investigation of psychiatric morbidity in 93 terminally ill cancer patients, focused solely on the Structured Clinical Interview for DSM Axis I disorders. The authors chose not to administer the interview for Axis II (personality) disorders, considering the patients too ill to undergo the additional assessment.

The prevalence of personality disorders was also not considered in a comprehensive literature review conducted by Walker [13] on mental disorders in patients with cancer in low- and lower-middle–income countries.

However, with patients now living many years after their initial diagnosis, often not completely cancer-free but undergoing ongoing life-saving treatments and regularly seeing the same doctors, personality functioning has become a crucial factor to consider and address properly in their care.

The emerging approach should be a personality-focused paradigm, as understanding the mechanisms through which personality is expressed, both in normal and pathological ways, is fundamental for the multidisciplinary team: the evolving nature of the disease makes it more beneficial to focus on personality functioning rather than merely symptoms, also in projecting proper clinical interventions.

Consistent with the Diagnostic and Statistical Manual of Mental Disorders [9,10,11], the distinguishing component of personality disorders is a pervasive pattern of maladaptive traits and behaviors that are relatively stable and emerge in early adult life. These traits and behaviors deviate from the expectations of the culture and lead to substantial distress and social dysfunction. The DSM-5 [11] classifies personality disorders into three categories or clusters: (A) odd or eccentric; (B) dramatic, emotional, or erratic; (C) anxious or fearful.

Cluster A includes paranoid, schizoid, and schizotypal personality disorders. The hallmark of these personality styles is the experience of pervasive social discomfort. Cluster A patients may make angry accusations and withdraw from staff, making staff feel misunderstood and wrongly accused. On the other hand, Cluster A patients may be perceived as odd and requiring attention [14,15]. These interpersonal dynamics can interfere with the establishment of a therapeutic alliance and may contribute to treatment avoidance or mistrust of healthcare professionals. As a result, staff may unintentionally disengage or respond defensively, further reinforcing maladaptive relational patterns and complicating the delivery of timely and appropriate care.

Cluster B includes antisocial, borderline, histrionic, and narcissistic personality disorders. Patients in Cluster B are typically characterized by high levels of interpersonal stress and shifting relationships, also with medical staff [14,15]. Their interactions may oscillate between idealization and devaluation, creating fluctuating emotional demands on clinicians and contributing to an unstable therapeutic relationship. Intense emotional expression, impulsivity, or entitlement may lead to boundary-testing behaviors, conflict with staff, or unrealistic expectations regarding treatment. These patterns can elicit strong countertransference reactions in healthcare providers, potentially resulting in burnout, frustration, or inconsistent engagement. If unaddressed, these relational difficulties may compromise adherence to medical treatment and the continuity of care.

Cluster C patients include avoidant, dependent, and obsessive–compulsive personality disorders. These patients are characterized by high levels of anxiety, fearfulness, and a strong need for control. With this kind of patient, staff may feel clumsy, overly protective, or as though their performance is being monitored and harshly judged [14,15]. These interpersonal patterns can foster excessive reassurance-seeking, reluctance to assume responsibility for treatment decisions, or avoidance of emotionally challenging topics. In the context of oncology, this may translate into hesitancy to engage in psychological support, rigid adherence to routines, or cancellation of sessions when anxiety becomes overwhelming. As a result, adherence to psychological treatments may be inconsistent, either due to fear of emotional exposure, fear of disappointing clinicians, or the perception that therapy threatens their sense of control. Recognizing these mechanisms can support clinicians in adopting a more structured, predictable, and validating approach to enhance therapeutic engagement and continuity of care.

It is well known that personality disorders can complicate the treatment of Axis I psychiatric disorders [16] and that these disorders are highly prevalent among patients who frequently use medical services [17]. The estimated prevalence of personality disorder varies between studies from 4.4% in Great Britain to 13.4% in Norway [18].

Given the growing number of long-term cancer survivors, it is numerically inevitable that the absolute prevalence of personality disorders within this population will increase, particularly when active screening is employed. Survivorship expands with the at-risk population, making the detection of pre-existing or comorbid personality pathology statistically more likely. Therefore, closer collaboration between clinical psychology and psycho-oncology should be promoted, particularly within training programs, as the demand for psychotherapy in psycho-oncology is likely to increase.

The label of ‘personality disorders’ may serve as a point of convergence, providing a shared language that fosters interdisciplinary collaboration between clinical psychologists, psychiatrists, and psycho-oncologists.

In this regard, focusing on personality disorders could also help further understand the themes of psychological distress screening in cancer patients, the number of referrals to specialist services, and the dropout phenomenon. There is a high dropout rate in cancer patients referred to mental healthcare [19], and researchers often interpret this data as a consequence of difficulties in attending psychological sessions due to illness and treatment.

We hypothesize that specific coping strategies, such as denial and avoidance, that are particularly representative of some personality disorders, may prevent patients from addressing their distress.

As regards the study of personality in psycho-oncology, researchers have often focused on specific traits, such as those outlined in Eysenck’s three-factor model of personality [20] and their impact on quality of life [21,22]. Some researchers have investigated the potential correlation between specific personality traits and particular cancer diagnoses. For example, Roso-Bas [23] in a study involving a cohort of 174 patients, found no significant differences in personality traits between lymphoma patients and the general cancer population, contrary to their initial hypothesis. Other researchers have concentrated on specific personality structures, such as Type D personality [24] or Type C personality [25], and their effects on health outcomes and psychological well-being [26]. Type C and Type D personalities are psychological constructs that describe certain consistent patterns of emotional and behavioral traits, often discussed in health psychology, especially in relation to illness, stress, and chronic disease outcomes like cancer and cardiovascular conditions.

In some studies, personality disorders have been treated as outcome variables, examining the impact of anxiety and depressive disorders on personality functioning; however psychological symptoms and personality disorders are often the result of a common etiology and coexist as pathoplastic factors [27].

Within the scope of health clinical psychology, which serves as a point of convergence between clinical psychology and psychosomatics, in the present systematic review we aim to focus on the prevalence of personality disorders in the cancer population, given their relevance to coping mechanisms in the face of chronic illness, their influence on health outcomes, and their impact on the dynamics with the multidisciplinary care team often over extended periods of time.

The dimensional–categorical approach to personality assessment and diagnosis, as outlined in Section 2 and Section 3 of the latest edition of the Diagnostic and Statistical Manual of Mental Disorders [9,10,11], was adopted for this purpose.

### Aims

Through this systematic literature review, we aim to evaluate studies that assess the prevalence of personality disorders, as defined by DSM Axis II, in cancer patients across all types of cancer and at every stage of the illness.

## 2. Materials and Methods

### 2.1. Protocol and Registration

This systematic review was conducted in accordance with the Preferred Reporting Items for Systematic Reviews and Meta-Analyses (PRISMA) guidelines [28], checklist and abstract in Tables S1 and S2. The review protocol was not prospectively registered in PROSPERO or any other systematic review registry.

### 2.2. Search Strategy to Identify Articles for This Analysis

To identify the relevant review literature, we chose a 45-year period (1980–2025). The main reason for choosing this period is that in 1980, the third version of the Diagnostic and Statistical Manual of Mental Disorders [9] for the first time defined personality disorders as inflexible, maladaptive, and causing significant impairment in functioning or subjective distress. In this edition, for the first time, five new personality disorders were added: schizotypal, narcissistic, borderline, avoidant, and dependent, as well as mixed personality disorder.

We searched three bibliographic databases: PubMed, Scopus, and Web of Science. For PubMed and Scopus, the following search string was used: ‘personality disorder OR personality pathology AND prevalence OR incidence AND cancer OR oncology AND patient OR individual.’ As related to PubMed, we used the following string inclusive of MESH: (“Personality Disorders” [Mesh] OR “personality disorder” [Title/Abstract:~3] OR “personality disorders” [Title/Abstract:~3] OR “personality disorder” [Title/Abstract:~3]) AND (“neoplasm” [Mesh] OR cancer or oncolog*”).

**Figure 1:**
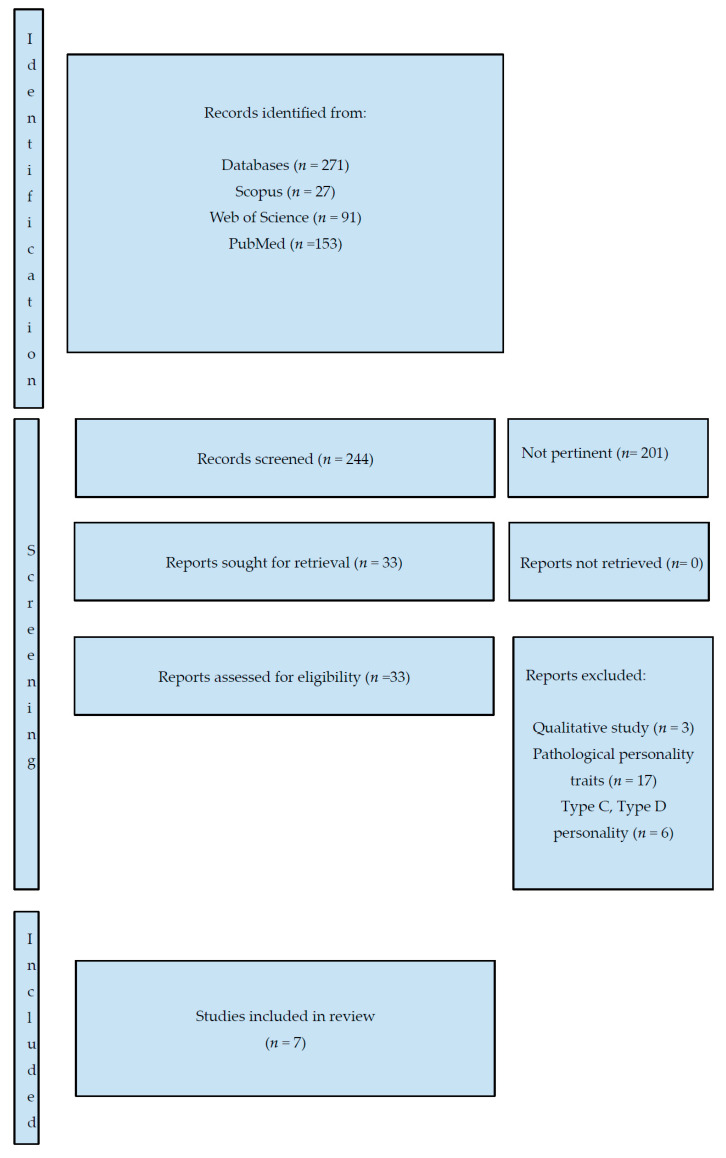
PRISMA 2020 flow diagram detailing the identification, screening, and inclusion of studies.

The search was limited to the English language and resulted in 271 articles (Figure 1). From these, we extracted seven relevant papers as described in Table 1. Due to the paucity of studies investigating the prevalence of personality disorders in cancer patients, we decided to include all types of studies: randomized controlled trials, cohort studies, cross-sectional studies, and longitudinal studies, while commenting on their methodological quality in a later section. We also included reviews and meta-analyses that incorporated relevant controlled studies.

P.A., I.M., and E.L. reviewed the titles and abstracts from the search to extract relevant papers. When they failed to reach consensus, M.C. was consulted to review the titles and abstracts of papers. We extracted only those papers with a major focus on personality disorders and cancer. We excluded papers with the following characteristics: (1) the focus was on personality traits and not on personality disorders, (2) the focus was on personality constructs such as Type C and Type D personality, and (3) we also excluded papers that focused on personality disorders and cancer but were qualitative in nature and did not report prevalence rates (*n* = 3).

### 2.3. Eligibility Criteria in Summary

Inclusion criteria:

- Studies reporting the prevalence of personality disorders
- Diagnosis of personality disorders based on DSM or ICD criteria
- Participants with a diagnosis of cancer (any type of cancer)
- Any type of studies
- Adult population (>18)
- Peer review original research articles

Exclusion criteria:

- Studies that do not provide prevalence data
- Diagnosis not based on validated DSM or ICD criteria
- Studies published before 1980

### 2.4. Quality of Papers

One goal of our review was to characterize the overall quality of the papers dealing with personality disorders and cancer. We did this by describing the quality of papers by using a systematic and comprehensive search strategy.

We referred to the Cochrane Handbook for Systematic Reviews Interventions [35]. A subjective assessment of methodological quality was performed by two authors (P.A., I.M.) using the following items, with each item rated as low, moderate, or high:

- likelihood of selection bias,
- study design,
- control of important confounders,
- data collection methods
- withdrawals and dropouts.

The overall rating is described in Table 1.

### 2.5. Quality Controls

We included only those papers that met specific methodological standards for assessing the prevalence of personality disorders in the cancer population. The studies were categorized into two broad groups: (1) personality disorders defined according to DSM or ICD classifications, and (2) dysfunctional personality patterns falling outside these diagnostic frameworks. Studies in the second category were excluded.

## 3. Results

### 3.1. Overall Results

Seven papers were deemed suitable for inclusion in our review.

We conducted a search covering a 45-year period, starting from the publication of the DSM-III [9], which was the first edition to conceptualize personality disorders in the way they are currently understood in general psychiatry and clinical psychology. We used two different search strings: one for Scopus and Web of Science, and another for PubMed. Scopus, Web of Science, and PubMed were all searched on 27 June 2025.

During the screening phase, P.A. noted that in some abstracts, the term ‘personality disorder’ was not explicitly mentioned, although it was specified within the full text of the article. For example, some abstracts referred to ‘other mental disorders,’ which, upon closer inspection, included personality disorders in the body of the paper. Consequently, P.A. and I.M. re-reviewed all selected papers, paying particular attention to this issue to ensure relevant studies were not overlooked. At the same time, a search on Google Scholar was conducted using the string ‘*prevalence of personality disorders AND cancer*’ in order to include results sections within the scope of the search. This yielded 1235 articles. We then cross-checked these results to ensure that no relevant articles were missed in our previous literature search.

We were specifically interested in including studies in which the authors focused on personality disorders. Consequently, we excluded all articles that investigated the prevalence of personality traits rather than diagnosable disorders. We also excluded studies on specific personality structures commonly examined in psychosomatic research, such as Type C and Type D personalities.

P.A., I.M. and E.L. independently screened the papers and discussed their initial selections during an online meeting lasting 4.5 h. Reaching consensus on the final seven papers required three additional working sessions of 2.5 h each. Each author created a table scoring the relevance of each selected article on a scale from 0 (not relevant) to 7 (most relevant). In the final online working session, only papers that received a score of 5 or higher were considered.

In cases where consensus could not be reached, M.C. was consulted to independently review the titles and abstracts of the relevant papers.

The main challenge arose in determining how to treat pathological personality traits, such as neuroticism or avoidance. Ultimately, the authors reached a consensus to include only studies that employed the DSM or ICD constructs of personality disorder. This decision was guided by the intention to promote a clinical health psychology perspective that would enable psycho-oncology mental health professionals to engage in more effective dialogue with general clinical psychologists and psychiatrists.

### 3.2. Selected Papers: Main Characteristics

The seven selected papers were published between 1983 and 2023. The diagnostic frameworks used for identifying personality disorders were various editions of the *Diagnostic and Statistical Manual of Mental Disorders* (DSM) (III, IV, and V) [9,10,11] and the *International Classification of Diseases* (ICD), including versions 9, 10, and 11 [36,37,38].

The selected studies were conducted in various parts of the world: three were from the United States [5,29,34], one from Italy [32], one from France [33], one from Turkey [31], and one from Egypt [30]. No studies from Oceania were available. Even though limited in number, with the sole exception of Oceania, studies have been conducted on every continent in the world.

In most cases, the instrument used to assess the presence of personality disorders was the Structured Clinical Interview for DSM Axis II Disorders (SCID-II) [39].

Regarding the cancer types represented in the samples, two studies included patients with all cancer diagnoses, while four focused exclusively on breast cancer. One study included a mixed sample comprising breast, lung, gastrointestinal, head and neck, and gynecologic cancers.

In terms of timing, the prevalence of personality disorders was assessed after diagnosis in two studies, after surgery in two others, and in the advanced stages of cancer in one. In the remaining two studies, patients at various stages of the illness were screened for mental disorders, including personality disorders.

Regarding the prevalence of personality disorders, in the study by Di Grezia [32], all breast cancer patients assessed at the time of diagnosis (*n* = 26) were found to meet the criteria for at least one personality disorder, as determined by the Structured Clinical Interview for DSM-IV Axis II. The high prevalence was not addressed nor commented on by the authors in the discussion section, which led us to rate the quality of the paper as low.

In the studies by Derogatis [5] and Miovic [34], the prevalence of personality disorders was found to be similar to that of the general population, approximately 3%. Derogatis investigated patients with various cancer diagnoses across different stages of illness, while Miovic focused specifically on patients with advanced cancer.

In the study by Anuk and colleagues [31], the prevalence of personality disorders was approximately 1.4%. The sample included cancer patients at different stages of illness, with the following distribution: breast cancer (31.1%), head and neck cancer (16.6%), gastrointestinal cancers (12.4%), lung cancer (11.1%), and gynecologic cancers (9.9%).

In the retrospective study by Shamsunder et al. [29], which investigated the impact of psychiatric diagnoses on patient-reported satisfaction and quality of life in postmastectomy breast reconstruction, the prevalence of personality disorders was reported at 14.3%. However, the authors did not specify the instrument used to assess mental disorders, referring only to a general clinical evaluation. Also, in this case, we rated the study quality as low.

Abdel-Fadeel et al. [30] investigated the prevalence of personality disorders in a sample of 150 breast cancer patients following surgery. Using the Structured Clinical Interview for DSM-IV, the study reported the following prevalence rates: 10% for paranoid personality disorder, 18.7% for borderline personality disorder, 22% for obsessive–compulsive personality disorder, and 2% for personality disorder not otherwise specified. Of note, the SCID-II [39] screening questionnaire is an instrument with yes/no questions rather than a comprehensive diagnostic tool, designed to facilitate the more efficient completion of the structured clinical interview for personality disorders, in which interviewers query only those items rated “yes” on the screening questionnaire.

Brunault and colleagues [33] also investigated a sample of breast cancer patients after diagnosis and found that 33.3% met the criteria for a personality disorder. Specifically, 23.3% had a Cluster A diagnosis, 7.5% a Cluster B diagnosis, and 18.3% a Cluster C diagnosis.

They used the Vragenlijst vor Kenmerken van de Persoonlijkheid (Questionnaire for Personality Traits) [40], which is a Dutch self-report questionnaire designed to screen for personality disorders based on the diagnostic criteria of the DSM-IV.

## 4. Discussion

Research on psychiatric morbidity in cancer populations has traditionally focused on detecting the prevalence of DSM Axis I disorders [7,8].

In contrast, the study of personality disorders has been largely neglected. Several factors contributed to this gap: the malignant and often life-threatening nature of cancer diagnoses, a symptom-focused clinical approach, and educational programs primarily designed to offer counseling and psychological support, rather than to foster deeper psychotherapeutic interventions [41].

However, the profound changes in cancer survivorship over recent decades call for a paradigm shift, one that recognizes a chronic phase in the cancer trajectory [1].

This evolving landscape necessitates a rethinking of psychosocial care models in oncology [42], making the assessment and understanding of personality disorders an essential component of comprehensive psycho-oncological care.

Given that the prevalence of personality disorders is closely linked to adverse childhood experiences (ACEs) [43] and considering that ACEs are both correlated with poorer health outcomes [44] and reported at higher rates among cancer patients [45], we can reasonably expect a growing prevalence of personality disorders in cancer survivors who require psychological care as survival rate grows among the cancer population.

Based on this reflection, the aim of this review is to serve as a starting point for developing a research trajectory focused on personality functioning up to dysfunction within the context of personalized care for cancer patients.

On the other hand, it is now well recognized that individuals with personality disorders who are engaged in long-term care for chronic health conditions often present significant challenges for healthcare personnel [46]. Therefore, it is essential to disseminate relevant clinical evidence and knowledge related to personality disorders and their management within healthcare settings.

It is important to first acknowledge that the overall quality of publications investigating the prevalence of personality disorders in the cancer population is limited. This is often due to insufficient methodological rigor, such as unclear specification of the questionnaires used to assess prevalence, or the lack of quantitative or mixed-method approaches. For instance, the SCID-II [39] screening questionnaire is an instrument with yes/no questions rather than a comprehensive diagnostic tool. While it is a well-validated and widely used instrument, its exclusive use may limit the generalizability of findings. As a clinician-administered interview, it may be influenced by interviewer bias and requires substantial training and time, which can affect consistency across studies. Furthermore, reliance on a single tool may also contribute to variability in reported prevalence and limit comparisons with studies using alternative diagnostic instruments or self-report measures. A valuable option would be to implement the use of the Personality Inventory for DSM-5 (PID-5) [47] as a standardized tool for assessing maladaptive personality traits in clinical and psycho-oncological settings.

Reported prevalence rates of personality disorders in cancer populations vary widely, ranging from very high estimates, 100% in the study of Di Grezia [32], to rates comparable to those found in the general population [5,31,34].

Importantly, these variations do not appear to be attributable to cultural factors or the geographic location where the studies were conducted: although limited in number, the studies are representative of almost every continent, apart from Oceania.

Consistent with broader trends in the psycho-oncology literature, the breast cancer population is the most frequently studied across various stages of illness, with a particular focus on the post-surgical phase, likely because it is the most commonly experienced stage among breast cancer patients [29,30,33].

### 4.1. Study Limitations

Despite its comprehensive scope, this review has certain limitations. One of the most significant relates to its objective: because the screening of personality disorders is largely overlooked in psycho-oncology, it is possible that relevant studies were missed, particularly if key information on personality disorders was presented only in the results section and not reflected in the title, abstract, or keywords, which are typically used for database searches. This likely reflects the fact that personality disorder assessment has rarely been the primary focus of studies in this field.

Through this review, we aim to highlight this gap and propose a new research direction that places greater emphasis on the role of personality in psycho-oncology.

The absence of multiple measures or triangulation increases the risk of both under- and overestimation, reducing comparability across studies.

### 4.2. Clinical and Research Implications

Efforts should be made to reach a consensus on the most appropriate quantitative measures and to identify which questionnaires are best suited to detect DSM or ICD-defined personality disorders in cancer populations, such as the one used by Brunault et al. in their study [33].

There is a clear need for studies using standardized and validated diagnostic instruments to assess personality disorders in the oncology population. Larger sample sizes are essential to increase statistical power and improve the reliability of prevalence estimates. Additionally, including a broader range of cancer types would enhance the generalizability of findings and allow for comparisons across different oncological contexts.

Alongside efforts to address methodological limitations in research, specialized education and training for mental health professionals in psycho-oncology are essential to ensure accurate assessment and effective management of personality pathology in this clinical setting.

Some suggestions in this regard are offered by Wynn in the Handbook of Psycho-Oncology [48], where the author provides guidance for healthcare providers working with patients affected by personality disorders, particularly emphasizing the importance of the time dimension and communication-related challenges.

We propose that the most effective approach to assessing personality disorders is the combined use of both the categorical and dimensional models presented in the DSM-5. In Section 2, the categorical approach remains the primary diagnostic framework, describing ten specific personality disorders grouped into three clusters, each with defined diagnostic criteria. In Section 3, clinicians and researchers are also introduced to an alternative dimensional model, which conceptualizes personality disorders in terms of impairments in personality functioning and maladaptive personality traits. The Personality Inventory for DSM-5 (PID-5) [47] may serve as a valuable screening tool within this integrative framework, supporting both categorical diagnosis and dimensional assessment [11].

## 5. Conclusions

Cancer has changed its face as an illness, and those who promote multidisciplinary psychosocial care should be aware of this transformation [49]. Focusing on personality functioning and its clinical counterpart, personality disorders may help clinicians better address the long-term needs of cancer patients. This perspective can also support the development of appropriate strategies to care effectively for the caregivers themselves.

## Figures and Tables

**Table 1:** Summary of the papers included in the systematic review, including their key characteristics and overall quality ratings.

Title	Reference	Region	Year of Publication	Number of Patients	Conceptualization of Personality Disorders	Method of Screening for Personality Disorders	Prevalence of Personality Disorders	Type of Cancer	Illness Phase	Type of Study	Overall Quality Rating of Papers
The Impact of Psychiatric Diagnoses on Patient-reported Satisfaction and Quality of Life in Postmastectomy Breast Reconstruction [29]	Shamsunder, M.G.	USA	2023	7414	ICD 9, ICD 10	No further specification of the clinical evaluation	up to 14.3%	Breast cancer	Post surgery	Retrospective study	Low
Personality, defense mechanisms, and psychological distress in women with breast cancer [30]	Abdel-Fadeel, N.A.	Egypt	2023	150	DSM-IV	Structured Clinical Interview for DSM-IV Axis II (SCID II)	10% paranoid; 18.7% borderline; 22% obsessive–compulsive; 2% not otherwise specified	Breast cancer	Post surgery	Cross-sectional study	Medium
The characteristics and risk factors for common psychiatric disorders in patients with cancer seeking help for mental health [31]	Anuk, D.	Turkey	2019	566	DSM-IV	Structured Clinical Interview for DSM-IV Axis II (SCID II)	1.4%	Breast cancer (31.1%); Lung cancer 11.1%; Gastrointestinal cancers 12.4%; Head and neck cancer 16.6%; Gynecologic cancer 9.9%	Various stages	Exploratory study with a retrospective chart-review design	Medium
Personality disorders and temperamental traits in patients with breast disease: preliminary results [32]	Di Grezia, G.	Italy	2016	29	DSM-IV	Structured Clinical Interview for DSM-IV Axis II (SCID II) version 2.0	All patients were reported to have a personality disorder	Breast cancer disease	Diagnosis	Prospective observational study	Low
Major depressive disorders, personality disorders, and coping strategies are independent risk factors for lower quality of life in non-metastatic breast cancer patients [33]	Brunault, P.	France	2016	120	ICD 10, DSM-IV	Vragenlijst voor Kenmerken van de Persoonlijkheid (Questionnaire for Personality Traits)	33.3% (Cluster A: 23.3%; Cluster B: 7.5%; Cluster C: 18.3%)	Breast cancer	3 months after diagnosis	Cross-sectional study	High
Psychiatric disorders in advanced cancer [34]	Miovic, M.	USA	2007	Not specified	DSM-IV	Not specified	2–3% (similar to the general population)	All cancer types	Advanced cancer	Review	Low
The prevalence of psychiatric disorders among cancer patients [5]	Derogatis, L.	USA	1983	215	DSM-III	Structured Clinical Interview for DSM Axis II (SCID II)	3%	All cancer diagnoses	Various phases of cancer	Observational	Medium

## Data Availability

No original data were generated for this systematic review. The data supporting the findings (e.g., list of included studies, search strategy, and data extraction tables) are available from the corresponding author (P.A.) upon reasonable request.

## Supplementary Materials

The following supporting information can be downloaded at: https://www.mdpi.com/article/10.3390/cancers17243901/s1, Table S1: PRISMA 2020 Checklist for Systematic Reviews; Table S2: PRISMA 2020 Extension for Abstracts for Systematic Reviews.
